# A Preclinical Systematic Review of Ginsenoside-Rg1 in Experimental Parkinson's Disease

**DOI:** 10.1155/2017/2163053

**Published:** 2017-03-13

**Authors:** Liang Song, Meng-Bei Xu, Xiao-Li Zhou, Dao-pei Zhang, Shu-ling Zhang, Guo-qing Zheng

**Affiliations:** ^1^Department of Neurology, The Second Affiliated Hospital and Yuying Children's Hospital of Wenzhou Medical University, Wenzhou, China; ^2^Department of Neurology, Zhengzhou People's Hospital, Zhengzhou 450003, China

## Abstract

To date, no drug has been proven to be neuroprotective or disease-modifying for Parkinson's disease (PD) in clinical trials. Here, we aimed to assess preclinical evidence of Ginsenosides-Rg1 (G-Rg1), a potential neuroprotectant, for experimental PD and its possible mechanisms. Eligible studies were identified by searching six electronic databases from their inception to August 2016. Twenty-five eligible studies involving 516 animals were identified. The quality score of these studies ranged from 3 to 7. Compared with the control group, two out of the 12 studies of MPTP-induced PD showed significant effects of G-Rg1 for improving the rotarod test (*P* < 0.01), two studies for improving the swim-score values (*P* < 0.01), six studies for improving the level of TH protein expression (*P* < 0.01), and two studies for increasing the expression of TH mRNA in the substantia nigra of mice (*P* < 0.01). The studies reported that G-Rg1 exerted potential neuroprotective effects on PD model through different mechanisms as antineuroinflammatory activities (*n* = 10), antioxidant stress (*n* = 3), and antiapoptosis (*n* = 11). In conclusion, G-Rg1 exerted potential neuroprotective functions against PD largely by antineuroinflammatory, antioxidative, and antiapoptotic effects. G-Rg1 as a promising neuroprotectant for PD needs further confirmation by clinical trials.

## 1. Introduction

Parkinson's disease (PD) is the second most frequent neurodegenerative disease after Alzheimer's disease characterized by the loss of dopamine-containing cells in the substantia nigra (SN) [1]. The clinical symptoms of PD are a wealth of motor symptoms and nonmotor symptoms. The treatment is divided into two directions: symptomatic therapy for motor symptoms and modifying the underlying disease process through neuronal protection or restoration. However, current treatments of PD are mainly symptomatic therapies and no treatment has yet been proven to be truly neuroprotective [2]. Dopamine replacement therapy (L-DOPA and dopamine agonists) is still the most effective symptomatic treatment of PD, but this treatment frequently induces therapy-related motor complications such as dyskinesia, choreoathetosis, and fluctuations in motor function [3]. Thus, a number of PD patients resort to various kinds of complementary or alternative medicine (CAM) to improve their motor and/or nonmotor symptoms [4]. Traditional Chinese medicine (TCM), as one of the most important parts in CAM, has played a vital role in the medical care of PD patients for thousands of years [5]. Ginseng, the root of* Panax* species (C.A. Meyer Araliaceae), is a well-known traditional Chinese herbal medicine that has been used for various kinds of diseases in China, Japan, and Korea for thousands of years and is still a popularly and worldwide used natural medicine in modern time [6]. The major pharmacologically active ingredients of ginseng are Ginsenosides and they are responsible for most of the activities of ginseng [7]. Ginsenosides are divided into two categories as follows: (1) the protopanaxadiol (PPD) type: Rb1, Rb2, Rb3, Rc, Rd, and Rg3; (2) the protopanaxatriol (PPT) type: Rg1, Re, Rf, and Rg2 [8]. It has been reported that Ginsenosides-Rg1 (G-Rg1) might have neuroprotective effects and little toxicity both in vitro and in vivo [9]. It also has beneficial effects on many neurological conditions, including the progressive neurodegenerative diseases such as PD [10]. The mechanisms of the neuroprotective effect of G-Rg1 include potentiating nerve growth factors, increasing anti-inflammation, antioxidation, and antiapoptosis, inhibiting excite toxicity and Ca^2+^ overinflux into neurons, maintaining cellular ATP levels, and preserving structural integrity of neurons [11]. However, no systematic review has been conducted to assess the effect of G-Rg1 on experimental PD models to date. Systematic review of all available evidence from animal experiments before clinical trials can provide us adequate interpretation of the limitations and potential of a novel treatment strategy [12]. Therefore, in the present study, we conducted a systematic review of all available animal studies to evaluate the preclinical evidence of G-Rg1 for experimental PD.

## 2. Methods

### 2.1. Search Strategy

Two trained researchers independently searched studies on the effects of G-Rg1 on PD from their inception to August 2016 in the following databases: PubMed, the Cochrane Database, Excerpta Medica (EMBASE), Chinese National Knowledge Infrastructure (CNKI), Wanfang database, and VIP Information Database. The following search terms were used: (Ginseng OR Ginsenoside OR Ginsenoside-Rg1 OR G-Rg1 OR Ginseng saponin) AND (Parkinson disease OR Parkinson's disease OR PD) in both English and Chinese.

### 2.2. Inclusion Criteria

Studies that were included met all of the following criteria: (1) all studies should test the effect of G-Rg1 on animal models of PD, regardless of language, blinding, or publication status; (2) in the treatment group, any intervention that used G-Rg1 for PD should be included irrespective of the frequency, dose, the method of injection, and intensity; (3) in the control group, animals were treated with normal saline or nothing.

### 2.3. Exclusion Criteria

Prespecified exclusion criteria were the following: (1) not reporting the efficacy of G-Rg1 on animal experiment of PD; (2) another neuroprotective agent being administered in the treatment group in addition to G-Rg1; (3) no control group; (4) reviews, case reports, abstracts, letters, comments, study protocol, editorials, and clinical guidelines; (5) duplicate publication.

### 2.4. Outcome Measurements

The primary outcome of the interest was the behavioral assessments, including rotarod test, pole test, wire suspension test, and the values of swim-score. Secondary outcomes were the number of Tyrosine Hydroxylase- (TH-) positive dopamine neurons in the substantia nigra pars compacta (SNpc), levels of TH protein expression in the SNpc, and the mechanisms of G-Rg1.

### 2.5. Data Extraction

Two investigators independently extracted information from each study, including (1) the first author's name and publication year; (2) individual data obtained for experimental animals including species, sex, number, weight, and anesthetic used; (3) experimental model; (4) information on treatment group including route of administration, dosage, and time for treatment; (5) data of control groups extracted as well as route of administration, dosage, and time of administration; (6) intergroup difference of each outcome measure; (7) outcome measures, including the behavioral exhibition of PD animal models, the number of TH neurons, and possible mechanisms of neuroprotective effects of G-Rg1 against PD. If outcomes were presented at different time points, data from the last time point were extracted. If the outcome data for meta-analysis were only expressed graphically or missing, we made attempt to contact authors for further information. When a response was not received, we used digital ruler software to measure the data from the graphs. We extracted data of mean value and standard deviation for each comparison from every study. Any disagreements were resolved through consultation with a corresponding author (Guo-qing Zheng).

### 2.6. Quality Assessment

The methodological quality of the included studies was assessed based on a nine-item modified scale from the Collaborative Approach to Meta-Analysis and Review of Animal Data from Experimental Studies (CAMARADES) [13]. The modified CAMARADES includes the following criteria: (1) peer reviewed publication; (2) control of temperature; (3) random allocation to groups; (4) blinded assessment of behavioral outcome; (5) use of anesthetic without significant intrinsic neuroprotective activity; (6) calculation of the sample size necessary to achieve sufficient power; (7) appropriate animal model which uses animals without relevant comorbidities (aged, diabetic, or hypertensive); (8) compliance with animal welfare regulations; (9) statement of potential conflict of interests. For quality assessment score, the interquartile range of score across studies was reported.

### 2.7. Statistical Analysis

We conducted statistical analysis using Cochrane's Review Manager (version 5.3) software. Data extracted from each study were considered as continuous data. WMD (weighted mean difference) is a standard statistic that measures the absolute difference between the mean values in two groups. Meanwhile, standardized mean difference (SMD) is also used as a summary statistic in meta-analysis when all the studies assess the same outcome but measure it in a variety of ways [14]. Heterogeneity among studies was estimated using Cochran's *Q* test (reported with *χ*^2^ value and *P* value) and *I*^2^ statistic. *I*^2^ values of 75, 50, and 25% correspond to high, medium, and low levels of heterogeneity, respectively; *I*^2^ values less than 50% indicated an acceptable degree of heterogeneity between studies [15]. Probability values of 0.05 were considered significant. Sensitivity analyses omitting each study at a time from the original analysis were conducted to verify our main results to be robust.

## 3. Results

### 3.1. Description of Studies

**Figure 1:**
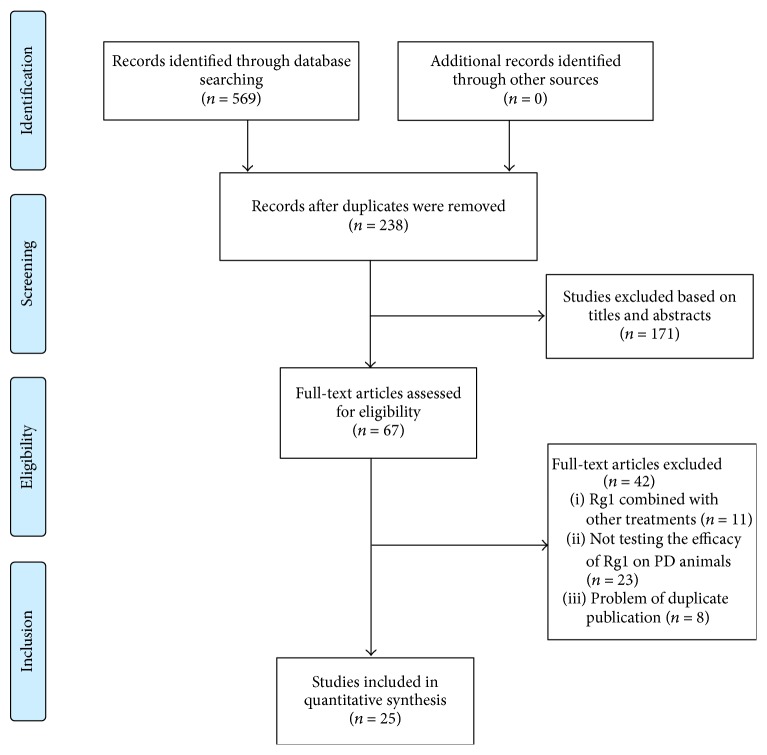
Summary of the process for identifying candidate studies.

We identified 569 potentially relevant articles from six electronic databases. After removing duplicates, 238 references remained. Through screening titles and abstracts, 171 studies were excluded. After full-text evaluation on the remaining 67 articles, 11 articles were removed because of combination with other treatment drugs in the experimental group; 23 articles were excluded because they did not test the efficacy of G-Rg1 on PD animals; 8 articles were excluded because of duplicate publication. Eventually, 25 eligible studies [16–40] were identified (Figure 1).

### 3.2. Study Characteristics

The 25 eligible studies included 516 animals from two species: 415 C57BL/6 mice and 111 ovariectomized Wistar rats. The weight of C57BL/6 mice varied from 16 g to 30 g, and the weight of ovariectomized Wistar rats varied from 200 g to 250 g. Eight articles [16–20, 35, 39, 40] were published in English academic journals and 17 articles [21–34, 36–38] were published in Chinese academic journals from 2001 to 2016. As for experimental animal model, twenty studies used 1-methyl-4-phenyl-1,2,3,6-tetrahydropyridine- (MPTP-) induced PD model [16–34, 39], 4 studies [35–38] used 6-hydroxydopamine (6-OHDA) induced PD model, and 1 study used lipopolysaccharide- (LPS-) induced PD model [40]. In terms of gender, five studies [25, 35, 37, 38, 40] used merely female animals and eighteen studies [16, 18–20, 22–24, 26–34, 36, 39] used merely male animals, while the remaining 2 studies [17, 21] did not report gender. Among 25 included studies, one study used ether cotton balls to induce anesthesia [33], two studies used urethane [17, 26], eleven studies used chloral hydrate [16, 23, 27, 28, 30, 32, 35–38, 40], four studies used pentobarbital sodium [18, 20, 31, 39], three studies did not report anesthesia [24, 29, 34], and the remaining four studies did not report the method of executing the animals [19, 21, 22, 25]. For all included studies, the intervention measures for experimental groups were injection with G-Rg1 before injection of MPTP, 6-OHDA, or LPS. Fifteen studies used behavioral assessments as primary outcomes [16, 17, 22–24, 26, 28, 29, 32–37, 39]. TH-positive dopamine neurons in the SNpc were observed in 16 studies [16–20, 24, 26, 28–32, 34, 35, 38, 39]. Nine studies reported the levels of TH protein expression [16, 18, 22–24, 29, 30, 32, 34]. Meanwhile, the indexes related to the mechanisms of G-Rg1 were used as outcomes as the anti-inflammatory activities in 10 studies [16–18, 21–23, 29, 33, 34, 40], antioxidant stress activities in 3 studies [19, 30, 36], and antiapoptosis in 11 studies [19, 20, 22–24, 26, 30–32, 35, 39]. The detailed characteristics of included studies are summarized in Table 1.

### 3.3. Risk of Bias in Included Studies

According to the nine-item modified CAMARADES checklist, the mean quality score of the 25 included studies was 5.12 (interquartile range: 4.75–6.0), with scores ranging from 3 to 7 (Table 2), of which one study [21] got 3 points; five studies [22, 24, 25, 29, 31] got 4 points; eleven studies [19, 20, 23, 26–28, 32–34, 36, 40] got 5 points; six studies [16, 17, 30, 37–39] got 6 points; and two studies [18, 35] got 7 points. All studies were published in peer reviewed journals and described random allocation to groups. None of the studies reported blinded assessment of behavioral outcome. Eighteen studies [16–18, 22–26, 28–30, 32–35, 37–39] reported the control of temperature. Six studies [19, 21, 22, 24, 25, 29] did not use anesthetic without significant intrinsic neuroprotective activity. All of the studies do not have formal sample size calculation. Thirteen studies [17–22, 25, 27, 30, 35–38] reported using of animals without relevant comorbidities (such as aging, diabetes, or hypertension). Three studies [21, 22, 25] did not report the compliance with animal welfare regulations. Seven studies [16–19, 35, 39, 40] stated potential conflict of interests.

### 3.4. Effectiveness

#### 3.4.1. Behavioral Assessments

**Figure 2:**
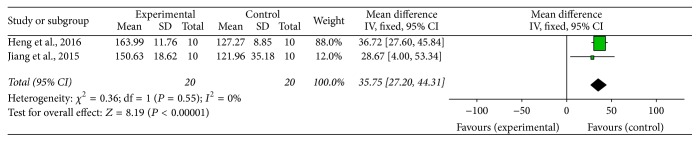
The forest plot: effects of G-Rg1 for improving the rotarod test compared with control group. Note: G-Rg1: Ginsenosides-Rg1.

**Figure 3:**
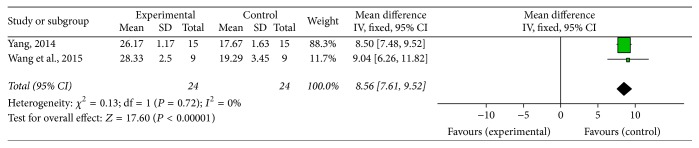
The forest plot: effects of G-Rg1 for improving the swim test compared with control group. Note: G-Rg1: Ginsenosides-Rg1.

Fifteen studies, including twelve MPTP-induced PD [16, 17, 22–24, 26, 28, 29, 32–34, 39] and three 6-OHDA induced PD [35–37] studies, used behavioral assessments as primary outcome measures. For the 12 studies on the motor dysfunction of MPTP-induced PD model, 2 studies [16, 17] provided clear data of rotarod test, 2 studies [22, 33] provided clear data of swim-score values, 4 studies [16, 17, 23, 39] provided graphical data of pole test, and the other 6 studies [24, 26, 28, 29, 32, 34] were descriptive studies without any data. Meta-analysis of 2 studies [16, 17] reported that the G-Rg1 group significantly improved rotarod test compared with MPTP-injected group (*n* = 40; WMD: 35.75; 95% CI: 27.20 to 44.31; *P* < 0.00001; heterogeneity: *χ*^2^ = 0.36; df = 1; *P* = 0.55; *I*^2^ = 0%) (Figure 2). Meta-analysis of 2 studies [22, 33] showed that the G-Rg1 group significantly improved the swim-score values compared with the MPTP-induced PD group (*n* = 48; WMD: 8.56; 95% CI: 7.61 to 9.52; *P* < 0.00001; heterogeneity: *χ*^2^ = 0.13; df = 1; *P* = 0.72; *I*^2^ = 0%) (Figure 3). Four studies [16, 17, 23, 39] indicated that mice treated with G-Rg1 spent less time descending the pole compared with mice treated with MPTP (*P* < 0.01 or *P* < 0.05 at different time point). There are two time durations that should be recorded in the pole test: one is the time it took the mouse to turn completely downward (T-turn) and the other one is the time it took the mouse to descend to the floor (T-total). But only one study recorded the two time durations; other studies did not clearly record them in detail. Meanwhile, the climbing pole time of mice in each study was conducted at different days. Owing to the above reasons, meta-analysis for this pole test could not be performed. The other 6 studies [24, 26, 28, 29, 32, 34] described that G-Rg1 group significantly improved the motor symptoms of PD induced by MPTP in mice, including the symptoms of thrilling, piloerection, raising tail, activity decrease, postural bradykinesia, and staggering gait but also failed to make a meta-analysis because they were just descriptive studies without any data. For the 3 studies on the motor dysfunction of 6-OHDA induced PD model, 2 studies [35–37] indicated that G-Rg1 group showed significant improvement in the rotational behavior in 6-OHDA-lesioned rats compared with control group; one study [17] reported that, in comparison with G-Rg1, mice treated with MPTP spent much more time reaching the platform during the wire suspension test (*P* < 0.05).

#### 3.4.2. The Number of TH-Positive Dopamine Neurons

**Figure 4:**
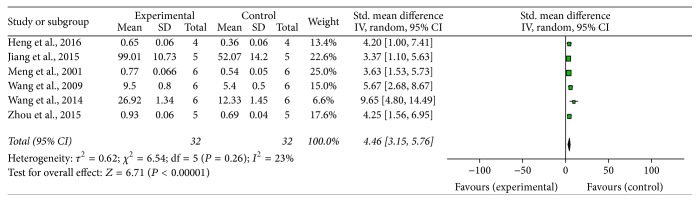
The forest plot: effects of G-Rg1 for improving the level of TH protein expression compared with control group. Note: G-Rg1: Ginsenosides-Rg1; TH: Tyrosine Hydroxylase.

**Figure 5:**
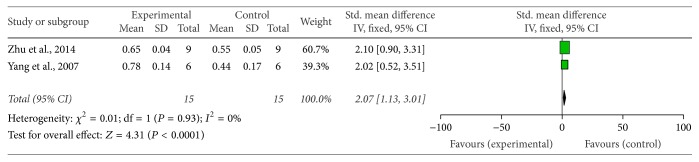
The forest plot: effects of G-Rg1 for improving number of TH mRNA compared with control group. Note: G-Rg1: Ginsenosides-Rg1; TH: Tyrosine Hydroxylase.

Sixteen studies, including fourteen MPTP-induced PD [16–20, 24, 26, 28–32, 34, 39] and two 6-OHDA induced PD [35, 38] studies, demonstrated the number of TH-positive dopamine neurons in the SNpc by immunohistochemistry analysis. Eleven out of 14 MPTP-induced PD studies provided raw data to make meta-analysis. Meta-analysis of 11 studies showed that G-Rg1 significantly improved the number of TH-positive neurons when compared with that in the MPTP-induced group (*n* = 180; WMD: 36.78; 95% CI: 35.27 to 38.28; *P* < 0.00001; heterogeneity: *χ*^2^ = 368.15; df = 10; *P* < 0.00001; *I*^2^ = 97%). Meanwhile, there was obvious heterogeneity for the analysis of TH-positive neurons between studies. Several factors were found to make significant influence on the outcome measure. When the authors counted the number of TH-positive neurons, the different types of microscopes, various magnification (such as ×10, ×40, ×100, and ×200), different sample drawing areas of the substantia nigra, different numbers of specimens of brain glass (such as 3 brain slices or 5 brain slices), different slices of brain tissue thickness (such as cut into 20 *μ*m and 30 *μ*m), and use of diverse anesthetics (such as chloral hydrate, pentobarbital sodium, and urethane) in different studies may contribute to this discrepancy. Thus, those reasons were considered as the potential sources of the heterogeneity. Seven studies [16–18, 22, 24, 32, 34] which reported the level of TH protein expression were qualified to perform a meta-analysis, and the random-effect model was applied for statistical analysis account for the heterogeneity (*n* = 82; SMD: 5.56; 95% CI: 3.56 to 7.56; *P* < 0.00001; heterogeneity: *χ*^2^ = 18.24; df = 6; *P* = 0.006; *I*^2^ = 67%) favouring G-Rg1 when compared with controls. We used sensitivity analyses omitting each study at a time from the original analysis. After removing 1 study [22] which was considered to be the potential source of the heterogeneity, the remaining 6 studies reported that G-Rg1 significantly improved the level of TH protein expression compared with control group (*n* = 64; SMD: 4.46; 95% CI: 3.15 to 5.76; *P* < 0.00001; heterogeneity: *χ*^2^ = 6.54; df = 5; *I*^2^ = 23%) (Figure 4). Two studies [23, 30] showed that G-Rg1 significantly increased the expression of TH mRNA in the substantia nigra of mice compared with the control group (*n* = 30; WMD: 2.07; 95% CI: 1.13 to 3.01;* P* < 0.00001; heterogeneity: *χ*^2^ = 0.01; df = 1;* P* = 0.93; *I*^2^ = 0%) (Figure 5).

#### 3.4.3. The Mechanisms of Neuroprotective Function of G-Rg1 in PD

**Figure 6:**
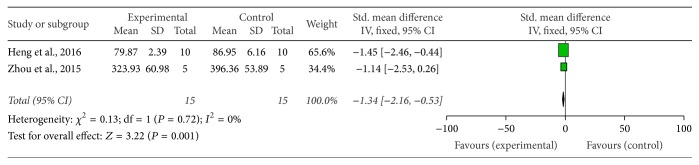
The forest plot: effects of G-Rg1 for decreasing the concentrations of IL-1*β* compared with control group. Note: G-Rg1: Ginsenosides-Rg1; IL-1*β*: cytokine interleukin-1*β*.

*Anti-Inflammatory Activities*. Ten studies [16–18, 21–23, 29, 33, 34, 40] reported the anti-inflammatory effect of G-Rg1 on the PD mice induced by MPTP (*n* = 7), 6-OHDA (*n* = 2), and LPS (*n* = 1) in the SNpc. Among them, only 2 studies [16, 18] reported the change of concentrations of cytokine interleukin-1*β* (IL-1*β*), whereas the other 8 studies failed to be pooled for analysis due to use of different anti-inflammatory indicators once or the absence of data. Meta-analysis of 2 studies [16, 18] showed that the concentrations of cytokine interleukin-1*β* (IL-1*β*) in the G-Rg1 groups significantly decreased compared with the control group (*n* = 40; SMD: −1.32; 95% CI: −2.02 to −0.62;* P* = 0.0002; heterogeneity: *χ*^2^ = 0.12; df = 1;* P* = 0.73; *I*^2^ = 0%) (Figure 6). Three studies [16, 18, 40] also showed that tumor necrosis factor-*α* (TNF-*α*), interferon-*γ* (IFN-*γ*), and IL-6 in the G-Rg1 groups significantly decreased compared with the control group (*P* < 0.01 or *P* < 0.05). Three studies [21, 22, 33] reported significant effects of G-Rg1 for decreasing the expression of erythropoietin-producing hepatocellular cell line such as EphA4, EphB6, and EphB1 compared with the control group in the SNpc (*P* < 0.01 or *P* < 0.05). One study [16] showed that G-Rg1 groups significantly decreased the expression of IBA-1 and GFAP proteins and the number of IBA-1- and GFAP-positive cells compared with the control group (*P* < 0.01). Two studies [29, 34] showed that G-Rg1 reduced COX-2 expression in the SN and might act on the P38 signaling pathway to protect the DA neurons in PD (*P* < 0.01).

*Antioxidant Stress*. Three studies including two MPTP-induced PD [19, 30, 36] and one 6-OHDA induced PD [36] studies reported the antioxidant stress effect of G-Rg1 on PD models. Two studies [19, 36] detected that G-Rg1 significantly increased glutathione (GSH) level and decreased total superoxide dismutase (T-SOD) activity and lactate dehydrogenase (LDH) levels in the SN compared with the control group (*P* < 0.01). The remaining study [30] showed significant effects of G-Rg1 for reducing the numbers of iron-staining cells compared with the control group (*P* < 0.01).

**Figure 7:**
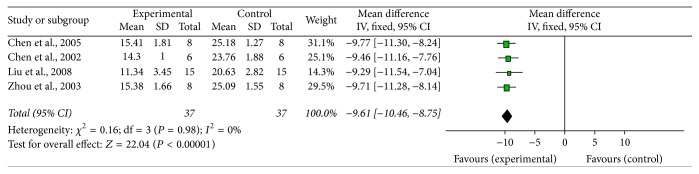
The forest plot: effects of G-Rg1 for improving TUNEL expression compared with control group. Note: G-Rg1: Ginsenosides-Rg1.

*Antiapoptosis*. Eleven studies [19, 20, 22–24, 26, 30–32, 35, 39] reported the effect of G-Rg1 against MPTP-induced (*n* = 10) or 6-OHDA (*n* = 1) induced apoptosis in mouse SN neurons. Four studies [19, 20, 26, 31] used the number of TUNEL-positive neurons as one of the indicators of antiapoptotic activities mechanisms. Meta-analysis of 4 studies [19, 20, 26, 31] showed that pretreatment with G-Rg1 remarkably decreased the TUNEL-positive neurons in the SN compared with the control (*n* = 74; WMD: −9.61; 95% CI: −10.46 to −8.75;* P* < 0.00001; heterogeneity: *χ*^2^ = 0.16; df = 3;* P* = 0.98; *I*^2^ = 0%) (Figure 7). The other 7 studies failed to be pooled for analysis due to lack of the data of TUNEL-positive neurons. Three studies [20, 30, 35] showed that G-Rg1 significantly increased the number of Bcl-2 and Bcl-xL cells compared with the control (*P* < 0.01 or *P* < 0.05). Six studies [20, 24, 26, 30–32, 39] reported that G-Rg1 remarkably decreased the number of caspase-3 positive cells in the SN compared with the control (*P* < 0.01 or *P* < 0.05). Three studies [22, 23, 31] reported that G-Rg1 dramatically decreased phospho-JNK and phospho-c-Jun protein expression compared with the control (*P* < 0.01 or *P* < 0.05).

## 4. Discussion

### 4.1. Summary of Evidence

Twenty-five studies with 516 animals were identified. This study found that G-Rg1 could improve the neurobehavioral abnormality and exert potential neuroprotective effects on PD model through different mechanisms such as antineuroinflammation, antioxidant stress, and antiapoptosis. However, we should treat the preclinical evidences cautiously because the methodological flaws undermine the validity of outcomes.

### 4.2. Methodological Considerations

This systematic review has a number of weaknesses. Firstly, animal studies with neutral or negative results may be more likely to remain unpublished and will be missed. Therefore, the effect size may be overstated. Secondly, our search strategy includes only Chinese or English databases, which may cause a certain degree of selective bias [41]. Thirdly, previous meta-analyses have suggested that animal studies that are less rigorously designed may overestimate treatment effects [42]. In the present study, all the studies failed to mention the blinded assessment of behavioral outcome. It may lead to performance bias and detection bias [43]. Sufficient size is essential to determine the efficacy of a new therapy or drug [44]. No study reported the calculation of the sample size that was necessary to achieve sufficient power, which indicated the lack of statistical power to ensure suitable estimation of the therapeutic effect [45]. Finally, the results from individual studies were inconsistent, and most of the studies used the graph rather than original data to present the outcomes. Therefore, we could not synthesize these data into the quantity.

### 4.3. Possible Neuroprotective Mechanism

The possible mechanisms of neuroprotective activity of G-Rg1 in PD are summarized as follows. (i) Inhibiting oxidative stress: high reactive iron levels can yield excess hydrogen peroxide and other reactive oxygen species (ROS), which will lead to mitochondrial dysfunction and increased dopamine metabolism. G-Rg1 could reduce the number of iron-staining cells in the SN of MPTP treated mouse [30] and showed protective effect. As one of the most important antioxidant molecules, GSH could clear H_2_O_2_ and prevent its reaction with iron to form the highly reactive ^*∙*^OH radical in the Fenton reaction. The present study showed that pretreatment with G-Rg1 could protect antioxidant defense system through attenuating the loss of GSH and increasing activity of T-SOD (including Cu/Zn-SOD and Mn-SOD) following MPTP treatment [19]. (ii) Inhibiting neuroinflammation: animal, human, epidemiologic, and therapeutic studies all revealed that the neuroinflammatory cascade plays a key role in the pathogenesis of PD. Recent studies demonstrated that G-Rg1 notably decreased neuroinflammation levels in the SNpc induced by MPTP. G-Rg1 could decrease the level of IBA-1, GFAP, EhpA, and EhpB protein expression, IBA-1, GFAP, EhpA, and EhpB positive cells, phosphorylated p38, COX-2, and PGE2 proteins, TNF-*α*, IL-1*β*, and the oligomeric*α*-synuclein expression in the SNpc [16–18, 21, 22]. (iii) Decreasing toxin-induced apoptosis: the protective effect of G-Rg1 against neurons apoptosis was related to enhancing Bcl-xL immunoreactive cells, Bcl-2 expression, TH^+^ neurons, reducing the level of caspase-3 cells, Bax, TUNEL neurons, and iNOS expression, and preventing c-Jun NH2-terminal kinase (JNK) signaling cascade [19, 20, 24, 26, 30, 31]. Therefore, G-Rg1 exerts beneficial effects on multiple aspects of the pathophysiology in PD.

### 4.4. Implications

It is well known that animal experiments have contributed to our understanding of mechanisms of diseases, but the translation of preclinical experiment which results in a prediction of the effectiveness of treatment strategies in clinical trials is still challenging [46, 47]. Previous studies [13] suggested that the quality of the research design is an important factor affecting the outcome. The main causes for the failure of translation of animal studies to human clinical trials include inadequate animal data and overoptimistic conclusions about efficacy drawn from methodologically flawed animal studies. Thus, it is essential to improve the methodological standards in the design, execution, and reporting of preclinical PD studies in the future.

Quantitative and statistical analysis of Ginsenosides in plasma indicates that PD type exhibits higher concentration and longer half-life than PT type [48]. Due to the low membrane permeability, active biliary excretion, and biotransformation, the oral bioavailability of G-Rg1 is very low [49–51]. After an oral administration of G-Rg1, the experiment in rats indicated that the area under the curve of G-Rg1 is 28.93 *μ*g·h·L^−1^ and the mean value of half-life is 15.26 hours. The peak concentration is 7.15 *μ*g·L^−1^, while *T*_max_ is 2.19 hours. In clinic, the use of ginseng for the suggestive symptoms of PD could date back to 1623–1670 AD recorded in Yizong Jiren Bian (Compiled Texts on Self Duty of Medicine) by Gao Gufeng who discussed the pathogenesis of tremor syndrome in the chapter shiver, shake, tremble: “Pathogenesis is mainly due to deficiency of Qi and Blood. The bones and muscles could not get enough nourishment, causing tremble that could not be controlled.” Ginseng Tonic Decoction should be used for treatment to invigorate Qi and Blood [5]. In fact, ginseng was one of most commonly used herbs for tremor syndrome from the Han Dynasty to the end of the Qing Dynasty (206 BC–1911 AD) in China by using the frequency statistics according to 232 prescriptions involving 193 herbs and 2529 total frequency of herbs [5]. In modern time, several clinical studies have been conducted to assess the efficacy and safety of ginseng prescription for PD, and the results indicated that ginseng prescriptions could significantly ameliorate the motor symptoms and improve the quality of life [52, 53]. However, no clinical study of G-Rg1 for PD has been yet conducted. In the present study, the findings indicated that G-Rg1 exerted potential neuroprotective functions against PD and its mechanisms are involved with on multiple aspects of the pathophysiology in multiple PD models. Thus, G-Rg1 may be a promising candidate neuroprotectant from bench to bedside. In addition, high-quality randomized controlled trials (RCTs) and a systematic review of those RCTs are commonly regarded the highest level of evidence in judging the treatment efficacy and safety of interventions [54]. Given the huge gap between the animal studies and the clinical trials, seeking and developing innovative neuroprotectants and further rigorous RCTs are urgently needed. In the present study, the findings indicated that G-Rg1 exerted potential neuroprotective functions against PD and its mechanisms are involved with multiple aspects of the pathophysiology of PD. Thus, G-Rg1 may be a promising candidate neuroprotectant from bench to bedside.

## 5. Conclusion

G-Rg1 exerted potential neuroprotective functions against PD despite of the methodological flaws. In addition, we identified an important area, which is worthy of further study. G-Rg1 as a promising clinical candidate neuroprotectant for PD needs to be further confirmed by clinical trials.

## Figures and Tables

**Table 1:** Characteristics of included studies.

Study (years)	Species (*n*)	Weight	PD model	Anesthetic	Experimental group	Control group	Outcome measure (experimental: G-Rg1/control)	Intergroup difference (ID)
Heng et al., 2016	Male, C57BL/6 mice (10/10)	22–25 g	MPTP-induced PD	Chloral hydrate (400 mg/kg, ip)	G-Rg1 (10, 20, 40 mg/kg, ip) for 49 d + MPTP (25 mg/kg, ip) for 4 d	MPTP (25 mg/kg, ip) for 4 d	(1) Behavioral tests: rotarod test, pole test (2) Numbers of TH(+) cells ↑ (3) TH protein expression ↑ (4) GFAP and IBA-1 expression ↓ (5) IBA-1 and GFAP(+) cells ↓ (6) Concentrations of TNF-*α*, IL-1*β* ↓ (7) Oligomeric *α*-synuclein expression ↓	(1) *P* < 0.01 (2) *P* < 0.01 (3) *P* < 0.05 (4) *P* < 0.01 (5) *P* < 0.01 (6) *P* < 0.05

Jiang et al., 2015	C57BL/6 mice (10/10)	25–30 g	MPTP-induced PD	Urethane (Sigma) (1.5 g/kg)	G-Rg1 (10 mg/kg, ip) for 15 d + MPTP (30 mg/kg, ip) for 5 d	MPTP (30 mg/kg, ip) for 5 d	(1) Behavioral tests: rotarod test, pole test, wire suspension test (2) TH(+) neurons ↑, TH protein expression ↑ (3) *α*-Synuclein expression ↓	(1) *P* < 0.01, *P* < 0.05 (2) *P* < 0.01 (3) *P* < 0.05

Zhou et al., 2016	Male, C57BL/6J mice (10/10)	16–25 g	MPTP-induced PD	2% pentobarbital sodium (40 mg/kg)	G-Rg1 (5, 10, 20 mg/kg, ip) for 10 d + MPTP (30 mg/kg, ip) for 5 d	MPTP (30 mg/kg, ip) for 5 d	(1) Behavioral tests: pole test (2) Numbers of TH(+) neurons ↑ (3) The protein levels of Wnt-1 and *β*-catenin ↑; GSK-3*β* and p-GSK-3*β* ↓ (4) Caspase-3 expression ↓, Bcl-xL ↑ protein levels of Wnt-1 ↑ and *β*-catenin ↑; GSK-3*β* ↓ and p-GSK- 3*β* ↓	(1) *P* < 0.01 (2) *P* < 0.01 (3) *P* < 0.01 (4) *P* < 0.01

Zhou et al., 2015	Male, C57BL/6J mice (5/5)	16–25 g	MPTP-induced PD	2% pentobarbital sodium (40 mg/kg)	G-Rg1 (5, 10, 20 mg/kg, ip) for 10 d + MPTP (30 mg/kg, ip) for 5 d	MPTP (30 mg/kg, ip) for 5 d	(1) The TH(+) neurons, levels of TH protein expression ↑ (2) T cell subsets: CD3^+^CD4^+^ T cells ↑, CD3^+^CD8^+^ T cells ↓ (3) Concentrations of TNF-*α*, IFN-*γ*, IL-1*β*, and IL-6 ↓ (4) Microglial cells not activated ↓	(1) *P* < 0.01 (2) *P* < 0.01, *P* < 0.05 (3) *P* < 0.01 or *P* < 0.05

Chen et al., 2005	Male, C57BL mice (8/8)	18–22 g	MPTP-induced PD	NR	G-Rg1 (5, 10, 20 mg/kg, ip) for 8 d + MPTP (30 mg/kg, ip) for 5 d	MPTP (30 mg/kg, ip) for 5 d	(1) Numbers of TH(+) ↑ and Nissl(+) neurons ↑; TUNEL(+) neurons ↓ (2) GSH level ↑; T-SOD activity ↓ (3) Phospho-JNK ↓ and phospho-c-Jun protein levels ↓	(1) *P* < 0.01 (2) *P* < 0.01

Chen et al., 2002	Male, C57BL mice (6/6)	20 ± 2 g	MPTP-induced PD	2% pentobarbital sodium	G-Rg1 (2.5, 5, 10 mg/kg, ip) for 8 d + MPTP (30 mg/kg, ip) for 5 d	MPTP (30 mg/kg, ip) for 5 d	(1) TH(+) neurons ↑; TUNEL(+) neurons ↓ (2) Bcl-2 cells ↑; Bcl-xL cells ↑; Bax cells ↓ (3) Caspase-3 cells ↓ (4) iNOS cells ↓	(1) *P* < 0.01 (2) *P* < 0.01 (3) *P* < 0.01 (4) *P* < 0.01

Wei et al., 2015	C57BL/6 mice (9/9)	22–30 g	MPTP-induced PD	NR	G-Rg1 (10 mg/kg, ip) for 7 d + MPTP (20 mg/kg, ip) for 4 d	MPTP (20 mg/kg, ip) for 4 d	(1) EphA4 mRNA expression ↓ (2) EphA4 protein expression ↓	(1) *P* < 0.01 (2) *P* < 0.01, *P* < 0.05

Wang et al., 2015	Male, C57BL/6 mice (9/9)	22–30 g	MPTP-induced PD	NR	G-Rg1 (10 mg/kg, ip) for 8 d + MPTP (20 mg/kg, ip) for 5 d	MPTP (20 mg/kg, ip) for 5 d	(1) Behavioral tests: the swim-score ↑ (2) TH protein expression ↑ (3) P-c-Jun protein expression ↓ (4) Numbers of EphB1(+) cells ↓	(1) *P* < 0.05 (2) *P* < 0.05 (3) *P* < 0.05 (4) *P* < 0.05

Zhu et al., 2014	Male, C57BL/6 mice (9/9)	22–30 g	MPTP-induced PD	6% chloral hydrate (30 mg/kg)	G-Rg1 (10 mg/kg, ip) for 8 d + MPTP (20 mg/kg, ip) for 5 d	MPTP (20 mg/kg, ip) for 5 d	(1) Behavioral tests: pole test (2) TH mRNA ↑ (3) Ephrin-B2(+) cells ↓(4) P-c-Jun(+) cells ↓	(1) *P* < 0.05 (2) *P* < 0.05 (3) *P* < 0.05 (4) *P* < 0.01

Wang et al., 2014	Male, C57BL/6 mice (9/9)	25–30 g	MPTP-induced PD	Anesthetized	G-Rg1 (10 mg/kg, ip) for 8 d + MPTP (30 mg/kg, ip) for 5 d	MPTP (30 mg/kg, ip) for 5 d	(1) Behavioral exhibition (2) Number of TH(+), FLIP(+), FADD(+), and caspase-3(+) cells (3) Expression level of TH, FLIP, FADD, and caspase-3 protein	(1) Not found (2) *P* < 0.01 (3) *P* < 0.01

Yan et al., 2014	Female, C57BL mice (10/10)	20 ± 2 g	MPTP-induced PD	NR	G-Rg1 (10 mg/kg, ip) for 8 d + MPTP (30 mg/kg, ip) for 5 d	MPTP (15 mg/kg, ip) for 5 d	(1) DA ↑ (2) Number of TH neurons ↑	(1) *P* < 0.01 (2) *P* < 0.01

Liu et al., 2008	Male, C57BL/6 mice (15/15)	18–23 g	MPTP-induced PD	20% urethane	G-Rg1 for 8 d + MPTP (30 mg/kg, ip) for 5 d	MPTP (30 mg/kg, ip) for 5 d	(1) Behavioral exhibition (2) Number of TH(+) cells ↑ (3) Number of caspase-3(+) cells ↓ (4) Number of TUNEL(+) cells ↓ (5) Caspase-3 protein ↓	(1) Not found (2) *P* < 0.05 (3) *P* < 0.05 (4) *P* < 0.05 (5) *P* < 0.05

Yang et al., 2009	Male, C57BL/6 mice (12/12)	17 ± 5 g	MPTP-induced PD	10% chloral hydrate (0.04 mg/kg)	G-Rg1 (70 mg/kg, ip) for 20 d + MPTP (30 mg/kg, ip) for 5 d	MPTP (30 mg/kg, ip) for 5 d	(1) Number of BrdU(+) cells ↑ (2) Number of Nestin(+) cells ↓ (3) BrdU+/Nestin+ cells ↑	(1) *P* < 0.01 (2) P < 0.01 (3) *P* < 0.05

Ji, 2008	Male, C57BL/6 mice (9/9)	25 ± 2 g	MPTP-induced PD	10% chloral hydrate (0.3 ml/kg)	G-Rg1 (5 mg/kg, ip) for 8 d + MPTP (30 mg/kg, ip) for 5 d	MPTP (30 mg/kg, ip) for 5 d	(1) Behavioral exhibition (2) Number of TH(+) cells ↑	(1) Not found (2) Not found

Wang et al., 2008	Male, C57BL/6 mice (10/10)	25–30 g	MPTP-induced PD	Anesthetized	G-Rg1 (10 mg/kg, ip) for 8 d + MPTP (30 mg/kg, ip) for 5 d	MPTP (30 mg/kg, ip) for 5 d	(1) Behavioral exhibition (2) Number of TH(+) cells ↑; number of COX-2 and PGE2(+) cells ↓ (3) Number of p-P38(+) cells ↓ (4) p-P38, COX-2, and PGE2 proteins ↓; TH proteins expression ↑	(1) Not found (2) *P* < 0.01 (3) *P* < 0.01 (4) *P* < 0.01

Yang et al., 2007	Male, C57BL/6 mice (6/6)	21 ± 2 g	MPTP-induced PD	Chloral hydrate	G-Rg1 (5 mg/kg, ip) for 8 d + MPTP (30 mg/kg, ip) for 5 d	MPTP (30 mg/kg, ip) for 5 d	(1) Number of TH(+) cells ↑; TH mRNA ↑ (2) DA, DOPAC, and HVA ↑ (3) Number of Fe(+) cells ↓ (4) Bcl-2 cells ↑, Bcl-2 mRNA ↑ (5) Caspase-3 and Bax(+) cells ↓, Bax and caspase-3 mRNA proteins ↓	(1) *P* < 0.01, *P* < 0.05 (2) *P* < 0.05 (3) *P* < 0.05 (4) *P* < 0.01, *P* < 0.05 (5) *P* < 0.01, *P* < 0.05

Zhou et al., 2003	Male, C57BL mice (8/8)	20 ± 2 g	MPTP-induced PD	2% pentobarbital	G-Rg1 (5, 10, 20 mg/kg, ip) for 8 d + MPTP (30 mg/kg, ip) for 5 d	MPTP (30 mg/kg, ip) for 5 d	(1) Number of TH(+) and Nissl(+) cells ↑ (2) Number of caspase-3(+) and TUNEL(+) cells ↓ (3) p-JNK and p-c-Jun protein expression ↓	(1) *P* < 0.01 (2) *P* < 0.01 (3) *P* < 0.01

Meng et al., 2001	Male, C57BL/6 mice (15/15)	25–30 g	MPTP-induced PD	10% chloral hydrate (0.3 ml/kg)	G-Rg1 (10, 20 mg/kg, ip) for 9 d + MPTP (30 mg/kg, ip) for 5 d	MPTP (30 mg/kg, ip) for 5 d	(1) Behavioral exhibition: pole test (2) Number of TH(+) cells ↑; number of GRP78(+), caspase-l2(+), and caspase-3(+) cells ↓ (3) TH protein expression ↑; GRP78, caspase-l2, and caspase-3 protein expression ↓	(1) Not found (2) *P* < 0.01 (3) *P* < 0.01

Yang, 2014	Male, C57BL/6 mice (15/15)	22–30 g	MPTP-induced PD	Ethyl ether	G-Rg1 (10 mg/kg, ip) for 9 d + MPTP (30 mg/kg, ip) for 7 d	MPTP (20 mg/kg, ip) for 7 d	(1) Behavioral assessments: the swim-score (2) EphB6 and Ephrin-B1 mRNA expression ↓ (3) EphB6 and Ephrin-B1 protein expression ↓	(1) *P* < 0.01 (2) *P* < 0.01 (3) *P* < 0.01

Wang et al., 2009	Male, C57BL/6 mice (15/15)	25–30 g	MPTP-induced PD	Anesthetized	G-Rg1 (10 mg/kg, ip) for 8 d + MPTP (30 mg/kg, ip) for 5 d	MPTP (30 mg/kg, ip) for 5 d	(1) Behavioral assessments (2) Number of TH(+) cells ↑; number of COX-2(+) cells ↓ (3) TH proteins expression ↑; COX-2 protein expression ↓	(1) Not found (2) *P* < 0.01 (3) *P* < 0.01

Xu et al., 2009	Female ovariectomized Wistar rats (12/12)	220–250 g	6-OHDA induced PD	Chloral hydrate (400 mg/kg)	G-Rg1 (10 mg/kg, ip) for 14 d + 6-OHDA (3.6 g/l, ip)	6-OHDA (3.6 mg/ml, ip)	(1) Reduced apomorphine-induced rotarod test (2) Number of TH(+) neurons ↑ (3) Gene expression of TH ↑ (4) The Bcl-2 protein and gene expression ↑	(1) *P* < 0.01 (2) *P* < 0.01 (3) *P* < 0.01 (4) *P* < 0.01

Jie, 2010	Male ovariectomized Wistar rats (10/10)	200–250 g	6-OHDA induced PD	10% chloral hydrate (3 ml/kg)	G-Rg1 (3 *μ*g/*μ*l, ip) + 6-OHDA (2 mg/ml, ip) for 7 d	6-OHDA (2 mg/ml, ip) for 7 d	(1) Behavioral tests: decreased apomorphine-induced rotarod test (2) SOD ↑, GSH level ↑, MDA ↓, LDH ↓	(1) Not found (2) *P* < 0.01

Xu et al., 2008	Female ovariectomized Wistar rats (6/6)	200–250 g	6-OHDA induced PD	8% chloral hydrate	G-Rg1 (10 mg/kg, ip) for 14 d + 6-OHDA (3.6 g/l, ip)	6-OHDA (3.6mg/ml, ip)	(1) Behavioral tests: decreased apomorphine-induced rotarod test (2) DA ↑, DOPAC ↑	(1) *P* < 0.01 (2) *P* < 0.01

Xu and Chen, 2007	Female ovariectomized Wistar rats (12/12)	200–250 g	6-OHDA induced PD	8% chloral hydrate	G-Rg1 (10 mg/kg, ip) for 14 d + 6-OHDA (3.6 mg/ml, ip)	6-OHDA (3.6 mg/ml, ip)	(1) Number of TH(+) neurons ↑ (2) TH gene expression ↑	(1) *P* < 0.01

Sun et al., 2016	Female ovariectomized Wistar rats (18/18)	250–300 g	LPS-induced PD	400 mg/kg chloral hydrate	G-Rg1 (10 mg/kg, ip) for 14 d + LPS (5.0 *μ*g, ip)	LPS 5.0 *μ*g dissolved in 2 *μ*l of 0.9% saline	(1) DA, DOPAC, and HVA ↑ (2) TNF-*α* and IL-1*β* ↓	(1) *P* < 0.01 (2) Not found

**Table 2:** Quality assessment of included studies.

Study	(1)	(2)	(3)	(4)	(5)	(6)	(7)	(8)	(9)	Score
Heng et al., 2016	√	√	√		√			√	√	6
Jiang et al., 2015	√	√	√		√		√	√	√	6
Zhou et al., 2015	√	√	√		√		√	√	√	7
Chen et al., 2005	√		√				√	√	√	5
Chen et al., 2002	√		√		√		√	√		5
Wei et al., 2015	√		√				√			3
Wang et al., 2015	√	√	√				√			4
Zhu et al., 2014	√	√	√		√			√		5
Wang et al., 2014	√	√	√					√		4
Yan et al., 2014	√	√	√				√			4
Liu et al., 2008	√	√	√		√			√		5
Yang et al., 2009	√		√		√		√	√		5
Ji, 2008	√	√	√		√			√		5
Wang et al., 2008	√	√	√					√		4
Yang et al., 2007	√	√	√		√		√	√		6
Zhou et al., 2003	√		√		√			√		4
Meng et al., 2001	√	√	√		√			√		5
Yang, 2014	√	√	√		√			√		5
Wang et al., 2009	√	√	√		√			√		5
Xu et al., 2009	√	√	√		√		√	√	√	7
Jie, 2010	√		√		√		√	√		5
Xu et al., 2008	√	√	√		√		√	√		6
Xu and Chen, 2007	√	√	√		√		√	√		6
Zhou et al., 2016	√	√	√		√		√	√		6
Sun et al., 2016	√		√		√		√	√		5

Note: (1) peer reviewed publication; (2) control of temperature; (3) random allocation to groups; (4) blinded assessment of behavioral outcome; (5) use of anesthetic without significant intrinsic neuroprotective activity; (6) calculation of the sample size necessary to achieve sufficient power; (7) appropriate animal model (aged, diabetic, or hypertensive); (8) compliance with animal welfare regulations; (9) statement of potential conflict of interests.
